# Active and inactive forms of biotin synthase occur in *Heterodera glycines*

**DOI:** 10.21307/jofnem-2019-069

**Published:** 2019-10-25

**Authors:** Khee Man Kwon, Sadia Bekal, Leslie L. Domier, Kris N. Lambert

**Affiliations:** 1 Department of Crop Sciences, University of Illinois, Urbana, IL; 2 Department of Agricultural and Biological Engineering, University of Illinois, Urbana, IL; 3 United States Department of Agriculture – Agricultural Research Service, Urbana, IL; 4 Department of Plant Pathology and Center for Applied Genetic Technologies, University of Georgia, Athens, GA

**Keywords:** Biochemistry, Biotin, Biotin synthase, Dethiobiotin, *Heterodera glycines*, Horizontal gene transfer, Soybean cyst nematode, Vitamin B_7_

## Abstract

*Heterodera glycines*, the soybean cyst nematode (SCN), is a plant-parasitic nematode capable of manipulating host plant biochemistry and development. Many studies have suggested that the nematode has acquired genes from bacteria via horizontal gene transfer events (HGTs) that have the potential to enhance nematode parasitism. A recent allelic imbalance analysis identified two candidate virulence genes, which also appear to have entered the SCN genome through HGTs. One of the candidate genes, *H. glycines* biotin synthase (*HgBioB*), contained sequence polymorphisms between avirulent and virulent inbred SCN strains. To test the function of these *HgBioB* alleles, a complementation experiment using biotin synthase-deficient *Escherichia coli* was conducted. Here, we report that avirulent nematodes produce an active biotin synthase while virulent ones contain an inactive form of the enzyme. Moreover, sequencing analysis of *HgBioB* genes from SCN field populations indicates the presence of diverse mixture of *HgBioB* alleles with the virulent form being the most prevalent. We hypothesize that the mutations in the inactive *HgBioB* allele within the virulent SCN could result in a change in protein function that in some unknown way bolster its parasitic lifestyle.

The soybean cyst nematode (SCN), *Heterodera glycines*, is an obligate plant parasite that poses a serious threat to soybean production worldwide. It is the most damaging pest of soybean in the USA and is estimated to cause more yield loss than any other disease (Koenning and Wrather, 2010; Wrather *et al.*, 2010; Allen *et al.*, 2017). Yield losses are attributed to the fact that this nematode injects a complex mixture of proteins and metabolites into the plant root cells (Niblack *et al.*, 2006) and transforms them into a metabolically active feeding structure called a syncytium (Hussey, 1989). The syncytium becomes a nutrient sink where the nematode feeds throughout its life cycle (Jones, 1981; Endo, 1998). This disrupts the plant’s vascular system and prevents normal functioning of the root system leading to lower soybean yields.

What makes matters worse is the nematode’s ability to persist in soils for 10 or more years even in the absence of a soybean host (Riggs, 2004). The most common and practical control strategy for SCN is to plant resistant cultivars and rotate to non-host crops (Niblack *et al.*, 2006). Although the management of SCN has been initially successful thanks to the use of resistant cultivars, the repeated use of the same resistance source has selected for virulent nematode populations that can reproduce on resistant cultivars (Niblack and Riggs, 2004; Mitchum *et al.*, 2007; Niblack *et al.*, 2008; Mitchum, 2016; McCarville *et al.*, 2017). Understanding how SCN evade or suppress host plant resistance at the molecular level may allow the development of more effective sources of SCN-resistant soybean.

To this end, tremendous effort was put in the past decades to understand the complex interactions between the nematode and its host plant. The majority of studies on plant-parasitic nematode-host interactions were mainly focused on discovering genes that code for secreted effector proteins originating from the esophageal glands involved in manipulating host plant biochemistry, development, and defenses (Hussey, 1989; Smant *et al.*, 1998; Lambert *et al.*, 1999; Davis *et al.*, 2000). Not all nematode secretory proteins, however, originate from the esophageal glands and some of the genes that encode secretory proteins can act as either virulence or avirulence genes. An esophageal gland-secreted protein/enzyme chorismate mutase (CM) is thought to suppress host defense by lowering the levels of chorismate-derived compounds required for plant defense (Lambert *et al.*, 1999; Doyle and Lambert, 2003). Some SCN CM genes contain polymorphisms correlating with virulence on resistant soybean cultivars (Bekal *et al.*, 2003; Lambert *et al.*, 2005), also implicating these genes in SCN virulence. Similarly, a cuticle-secreted lipid-binding protein Gp-FAR-1 from *Globodera pallida* may be able to inhibit the host’s jasmonic acid signaling pathway (Prior *et al.*, 2001). In contrast, the SPRYSEC effector protein Gp-RBP-1 secreted from an esophageal gland in *G. pallida* (Sacco *et al.*, 2009), the venom allergen-like effector protein Gr-VAP1 secreted from an esophageal gland in *G. rostochiensis* (Lozano-Torres *et al.*, 2012), MAP-1 secreted from amphids in *Meloidogyne incognita* (Semblat *et al.*, 2001), and a SNARE-like protein (*HgSLP-1*) secreted from an esophageal gland in *H. glycines* (Bekal *et al.*, 2015) appear to act as avirulence genes. Although not a secreted protein, Cg-1 from *M. javanica* is also thought to function as an avirulence gene (Gleason *et al.*, 2008).

Some of the secreted proteins are closely related to bacterial proteins, suggesting that they have been acquired from bacteria through horizontal gene transfer events (HGTs) (Smant *et al.*, 1998; Lambert *et al.*, 1999; Davis *et al.*, 2000; Bird *et al.*, 2003; Bekal *et al.*, 2015). While these studies focusing on secreted proteins and HGT candidates have been productive for understanding the molecular basis of nematode-host interactions, non-secreted proteins, enzymes, and metabolites have been overlooked; hence, horizontally transferred genes coding for non-secreted proteins/enzymes with bacterial homology could also play a critical role in SCN biology, parasitism, and (a)virulence.

Studies on non-secreted plant-parasitic nematode proteins have been few in number until Craig *et al*. (2008, 2009) showed that a certain group of non-secreted HGT proteins can include enzymes involved in vitamin B_1_, B_5_, B_6_, and B_7_ biosynthesis and their corresponding salvage pathways. More recently, a whole genome allelic imbalance analysis, conducted for the purpose of identifying single nucleotide polymorphisms (SNPs) associated with SCN genes implicated in virulence, found two candidate virulence genes, *HgSLP-1* and biotin synthase (*HgBioB*), both of which have been acquired through HGTs (Bekal *et al.*, 2015). *HgBioB* is a non-secreted protein/enzyme responsible for catalyzing the conversion of dethiobiotin (desthiobiotin or DTB) to biotin (vitamin B_7_). Furthermore, *HgBioB* contained SNPs that generated amino acid polymorphisms between avirulent and virulent inbred SCN strains, suggesting the allelic forms of *HgBioB* may have different activity (Bekal *et al.*, 2015).

We postulated that these sequence polymorphisms could alter *HgBioB* activity and play an important role in SCN parasitism or virulence. Because it seemed reasonable that a nematode’s ability to produce more biotin might counteract a plant resistance mechanism that restricts the nematode from accessing free biotin or its immediate precursor, we hypothesized that virulent nematodes, which can reproduce on resistant cultivars, might produce a more active form of *HgBioB* than their avirulent counterparts, which cannot parasitize the resistant hosts. To support or refute this hypothesis, we tested the function of the two *HgBioB* alleles to ascertain if any functional differences could be detected between the virulent and avirulent forms of the enzyme.

## Materials and methods

### Construction and cloning of recombinant plasmids containing *HgBioB*

Two different DNA fragments *HgBioB*-avr (avirulent; accession number MH251885) and *HgBioB*-vir (virulent; accession number MH251886) that code for *HgBioB* in inbred SCN strains TN10 (avirulent; Hg-type 0; grown on Roma tomato) and TN20 (virulent; Hg-type 1, 2, 3, 4, 5, 6, 7; grown on plant introduction 437654), respectively, were synthesized with codons optimized for *E. coli* expression (Life Technologies, Carlsbad, CA). Also, two different mutagenized forms of *HgBioB*-vir, *HgBioB*-vir-M1 (virulent, mutagenized allele 1; accession number MH251887) and *HgBioB*-vir-M2 (virulent, mutagenized allele 2; accession number MH251888), were prepared by generating (i) an alanine to proline mutation (M1) at amino acid position 24 (A24P); and (ii) a glutamine to arginine mutation (M2) at amino acid position 44 (Q44R), respectively. M1 was produced via site-directed mutagenesis following the manufacturer’s instructions for In-Fusion cloning (Clontech, Mountain View, CA), using *HgBioB*-vir as the template. A pair of inverse polymerase chain reaction (PCR) primers (Table 1), BioB-mut1-F and BioB-mut1-R, was designed to share a 15-bp homology with each other and include a single-base substitution to generate an A24P mutation. Unlike M1, M2 was prepared by custom-ordering the synthetic DNA fragment containing the desired Q44R mutation (Life Technologies).

**Table 1:** Oligonucleotides for plasmid construction and sequencing analysis.

Primer name	Sequence
BioB-F	5′-GAA GGA GAT ATA GAT ATG CCT CCG CCT ATT GGT AGC-3′
BioB-R	5′-TTA TGG AGT TGG GAT TTA CAG ATT CAG GGT CAC TTT TTC ATC GT-3′
BioB-mut1-F	5′-CCA TTT CCG GAA CTG ATT TTT CGT GCA CAG AAT GTT C-3′
BioB-mut1-R	5′-CAG TTC CGG AAA TGG CAG GCT AAA AAC GCT CAG TGC-3′
BioB-Vec-F	5′-ATC CCA ACT CCA TAA GGA TCC CTT G-3′
BioB-Vec-R	5′-ATC TAT ATC TCC TTC TTA AAG TTA AAC AAA ATT ATT TCT AGA TGT AGA TGT TAG CC-3′
BioB-flank-F1a	5′-GGA GAG GAA TGA TAT GAT GAA-3′
BioB-flank-R1a	5′-CAT CTT CTG CTT CTG TTC TG-3′

To perform In-Fusion cloning (Clontech), two pairs of PCR primers (Table 1) were designed for the insert and the vector so that they would share a 15-bp homology with each other. All four DNA fragments were amplified using the PCR primers BioB-F and BioB-R; and pBAD.LIC.8A cloning vector (Addgene plasmid No. 37501) was amplified with PCR primers BioB-Vec-F and BioB-Vec-R. All PCRs were done using an iProof HF Mastermix (Bio-Rad, Hercules, CA) following the manufacturer’s recommended PCR conditions. The resulting PCR products were gel-purified using a QIAquick Gel Extraction Kit (Qiagen, Valencia, CA), joined together using an In-Fusion HD Cloning Kit (Clontech), and transformed into Stellar Competent Cells (Clontech) following the manufacturer’s instructions. Cells containing successful transformants were screened on Luria-Bertani (LB) agar plates with ampicillin (100 mg/ml) and a single colony was picked to inoculate 5 ml of LB broth with ampicillin (100 mg/ml) which was incubated overnight at 37°C. Recombinant plasmid DNA was extracted from the overnight culture using a QIAprep Spin Miniprep Kit (Qiagen) and the resulting plasmids were sequenced for verification at the University of Illinois Roy J. Carver Biotechnology Center.

### Complementation of ΔbioB *Escherichia coli* with avirulent and virulent alleles of *HgBioB*

A kanamycin-resistant *E. coli* vitamin B_7_ auxotroph (K-12 Keio Collection: JW0758-1) was obtained from the Coli Genetic Stock Center at Yale University and used to prepare electrocompetent cells as described by Dower *et al*. (1988) and Hanahan (1983). Four different recombinant plasmids – pBAD.LIC.8A:: *HgBioB*-avr, pBAD.LIC.8A:: *HgBioB*-vir, pBAD.LIC.8A:: *HgBioB*-vir-M1, and pBAD.LIC.8A:: *HgBioB*-vir-M2 – were generated and independently transformed into the kanamycin-resistant ΔbioB mutant electrocompetent cells. For control purposes, an empty vector (pBAD.LIC.8 A) lacking any inserts was also transformed into the electrocompetent cells. The resulting transformants were screened on LB agar plates with ampicillin (100 mg/ml) and kanamycin (50 mg/ml), and a single colony was picked to inoculate 10 ml of LB broth with ampicillin (100 mg/ml) and kanamycin (50 mg/ml) for overnight incubation at 37°C. The overnight cultures of these four strains were thoroughly washed in liquid M9 minimal media to remove trace amounts of biotin that may be present, and then streaked onto M9 minimal media plates containing ampicillin (100 mg/ml), kanamycin (50 mg/ml), and either avidin or biotin. Plates containing avidin (1 mg/ml) were prepared to sequester trace amounts of biotin in the M9 media. Positive control plates were M9 media supplemented with biotin to a final concentration of 0.2 mM, and a negative control plate consisted of ΔbioB *E. coli* transformed with an empty pBAD.LIC.8A vector plated on M9 media lacking biotin. Some plates were also treated with 2% arabinose to induce *HgBioB* expression and monitor its potential effect on *E. coli* growth. All plates were initially incubated at 28°C for 48 hr, and then allowed to grow at room temperature for 10 d. *E. coli* growth was monitored daily.

### Nematode culture and DNA extraction of SCN field populations

A collection of soil samples containing diverse SCN field populations representative of the *H. glycines* diversity was randomly chosen and prepared for nematode culture. The majority of these soil samples was from Iowa, a kind gift from Dr Gregory Tylka at Iowa State University (Ames, IA), and some of them were from Illinois. At least two to three different field populations originating from the same geographical region (e.g., at least two to three field populations from the east central region) were randomly selected for cyst extraction. Cysts from each field population were collected as described by Niblack *et al*. (1993) and crushed over a 0.25-mm (60 mesh) sieve to release eggs. Eggs were suspended in 2% carboxymethyl cellulose solution for an easy delivery of an approximately equal number of eggs during inoculation. This egg suspension was used to inoculate soybean cv. Essex plants, previously pre-germinated and planted into 50-ml Falcon tubes with the absorbent wick described in Bekal *et al.* (2015). Inoculated plants were grown for 8 wk to allow eggs to hatch and infect the plants. The resulting cysts from each field population, approximately 2 to 200 cysts from each plant, were harvested in a 2-ml cryotube vial, and stored at −80°C until use. The cyst-containing vials were placed in a pre-cooled rack in liquid nitrogen and two stainless steel beads (3.2 mm diameter) were added into each vial. These frozen cysts were then freeze-fractured with a bead beater homogenizer and total nucleic acid was extracted using an RNeasy Mini Kit (Qiagen).

### Sequencing and statistical analysis of different SCN populations

In addition to 30 DNA samples from field populations, 4 DNA samples from inbred SCN strains – 1 avirulent (OP25) and 3 virulent inbred strains (OP20, OP50, and TN20) – were also included in the analysis. The OP strains were previously maintained as described in Dong and Opperman (1997) by Dr Terry Niblack (Ohio State University, Columbus, OH), and TN20 was maintained on PI 437654. From each extracted 50-μl DNA sample from a single population, 2.5 μl was used as a template for amplification using a GenomiPhi Kit (GE Healthcare, Piscataway, NJ) following the manufacturer’s protocols. The amplified DNA was treated with ExoSAP-IT (Affymetrix, Santa Clara, CA) for the degradation of primers and nucleotides that may interfere with downstream applications. A set of PCR primers (Table 1), BioB-flank-F1a and BioB-flank-R1a, flanking the polymorphic region of the *HgBioB* gene was designed for PCR amplification and subsequent sequencing analysis. All PCRs were done using a CloneAmp Hi-Fi PCR Premix (Clontech) following the manufacturer’s recommended conditions. The resulting PCR product was gel-purified using a QIAquick Gel Extraction Kit (Qiagen) and then sent for sequencing at the University of Illinois Roy J. Carver Biotechnology Center. The sequences were aligned to the original *HgBioB* sequence using Sequencher (Gene Codes Corporation, Ann Arbor, MI), and the relative chromatogram peak heights corresponding to amino acid positions 24 and 44 were measured with a 15-cm ruler on the computer screen to estimate the percentage of each polymorphic nucleotide(s).

Peak heights were statistically analyzed using both descriptive and inferential methods. First, a box plot was generated to compare the median values and understand the distributional characteristics of these alleles at both polymorphic regions, and then a Wilcoxon signed rank test was performed using the SAS Proc Univariate procedure (SAS Institute, Cary, NC) to test if the differences between avirulent and virulent alleles were statistically significant. For the signed rank test, a new variable ‘diff’ was set as the difference between the virulent and avirulent alleles (diff = vir − avir) at each amino acid for each SCN sample prior to running the Proc Univariate procedure on that variable. Finally, a Spearman rank-order correlation was conducted using the SAS Proc Corr procedure (SAS Institute) to calculate the coefficients for the relationship between the two variables, amino acid positions 24 and 44, for each set of virulent and avirulent pairs.

## Results

### Complementation of ΔbioB *E. coli* with avirulent and virulent alleles of *HgBioB*

We had observed differences in the predicted amino acid sequences between avirulent and virulent *HgBioB* alleles (Bekal *et al.*, 2015), and thus hypothesized that such differences could lead to an altered enzyme function. To test for *HgBioB* function, we opted to use genetic complementation of a BioB-deficient *E. coli* mutant. Specifically, we wanted to learn if amino acid(s) changes, P24A or R44Q, the only two amino acids that are different between the *HgBioB* alleles, would alter BioB enzyme function.

**Figure 1:**
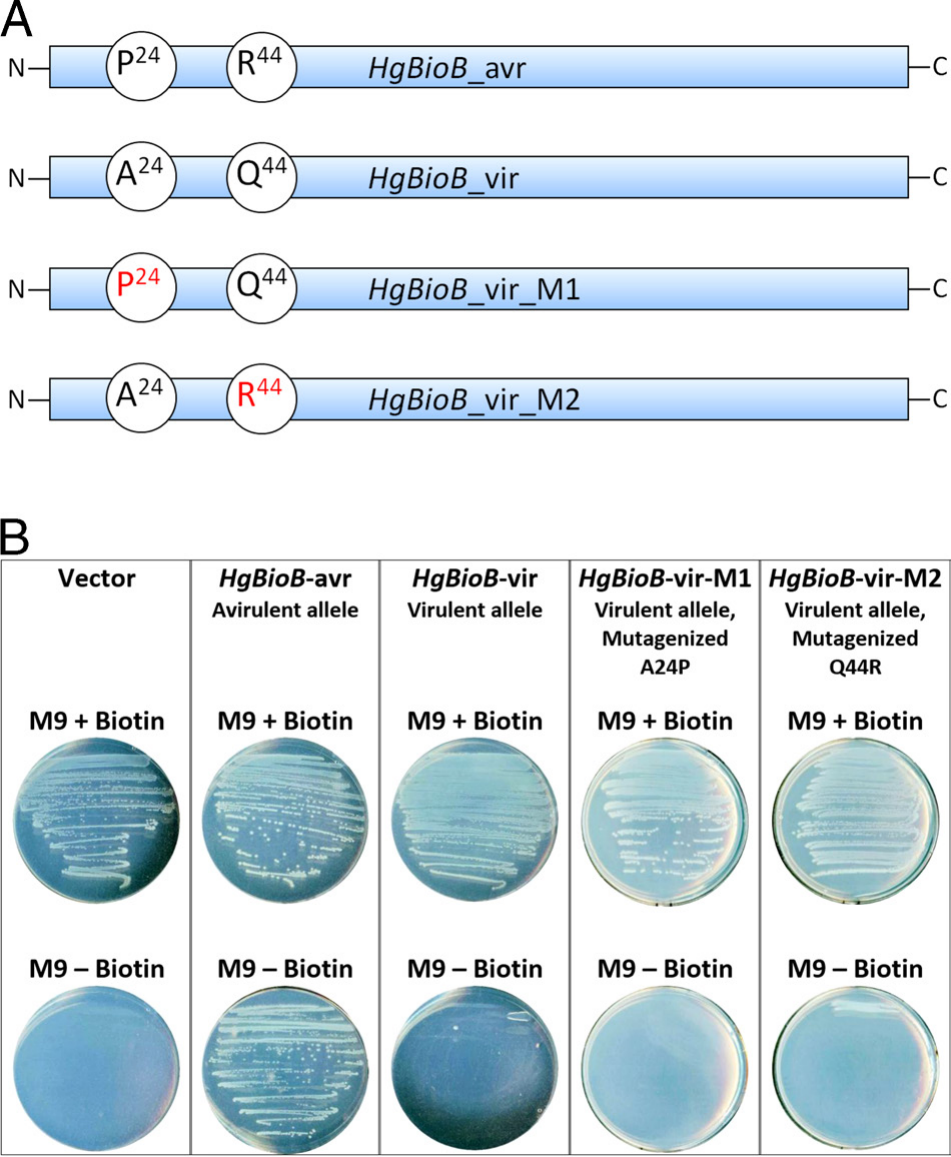
Complementation by expression of *HgBioB* in biotin synthase-deficient *E. coli* strain. (A) *HgBioB* construct diagrams showing polymorphic amino acids, from N-terminus to C-terminus, at positions 24 and 44. *HgBioB*-vir was mutagenized to produce *HgBioB*-vir-M1, by generating an alanine to proline mutation at position 24 (A24P); and *HgBioB*-vir-M2, by generating a glutamine to arginine mutation at position 44 (Q44R). (B) The expression of the vector control, *HgBioB*-avr, *HgBioB*-vir, and two mutagenized forms of the virulent allele *HgBioB*-vir-M1 and *HgBioB*-vir-M2 on minimal media with and without supplemental biotin.

To conduct this experiment, four plasmid constructs were generated to express the different forms of *HgBioB*; the virulent allele (*HgBioB*-vir, A24/Q44), the avirulent allele (*HgBioB*-avr, P24/R44), and the two single mutant alleles (*HgBioB*-vir-M1, P24/Q44; and *HgBioB*-vir-M2, A24/R44) (Fig. 1A). These two single mutants prepared from the virulent allele were included in the complementation studies to identify which amino acid location(s) were responsible for a functional enzyme activity. These plasmids, along with an empty vector control, were transformed into mutant *E. coli* lacking endogenous BioB function and were then allowed to grow on minimal agar with and without supplemented biotin.

Within 48 hr after streaking the *E. coli* containing the BioB constructs onto minimal media plates, all five biotin-supplemented plates (minimal media +biotin; positive controls) showed vigorous growth of *E. coli* as expected. Among the five plates lacking biotin (minimal media alone), however, growth only occurred for the bacteria containing the avirulent allele containing the P24/R44 amino acid combination (*HgBioB*-avr) (Fig. 1B). The other four constructs, *E. coli* carrying the empty vector (negative control), the virulent allele (*HgBioB*-vir), and the two mutagenized forms of the virulent allele (*HgBioB*-vir-M1 and *HgBioB*-vir-M2) failed to complement the growth of the mutant *E. coli* (Fig. 1B).

Our complementation test showed that avirulent SCN produces an active biotin synthase while virulent SCN contains an inactive form of the enzyme. Additionally, site-directed mutagenesis experiments confirmed that changing either of these two amino acids caused the *HgBioB* protein to become inactive, losing its ability to complement the mutant; therefore, a proline at position 24 (P24) and an arginine at position 44 (R44) are required for a functional BioB activity (Fig. 1A,B). This experiment was repeated and produced the same results.

### Sequencing, statistical analysis, and confirmation of SNPs from SCN field populations

**Figure 2:**
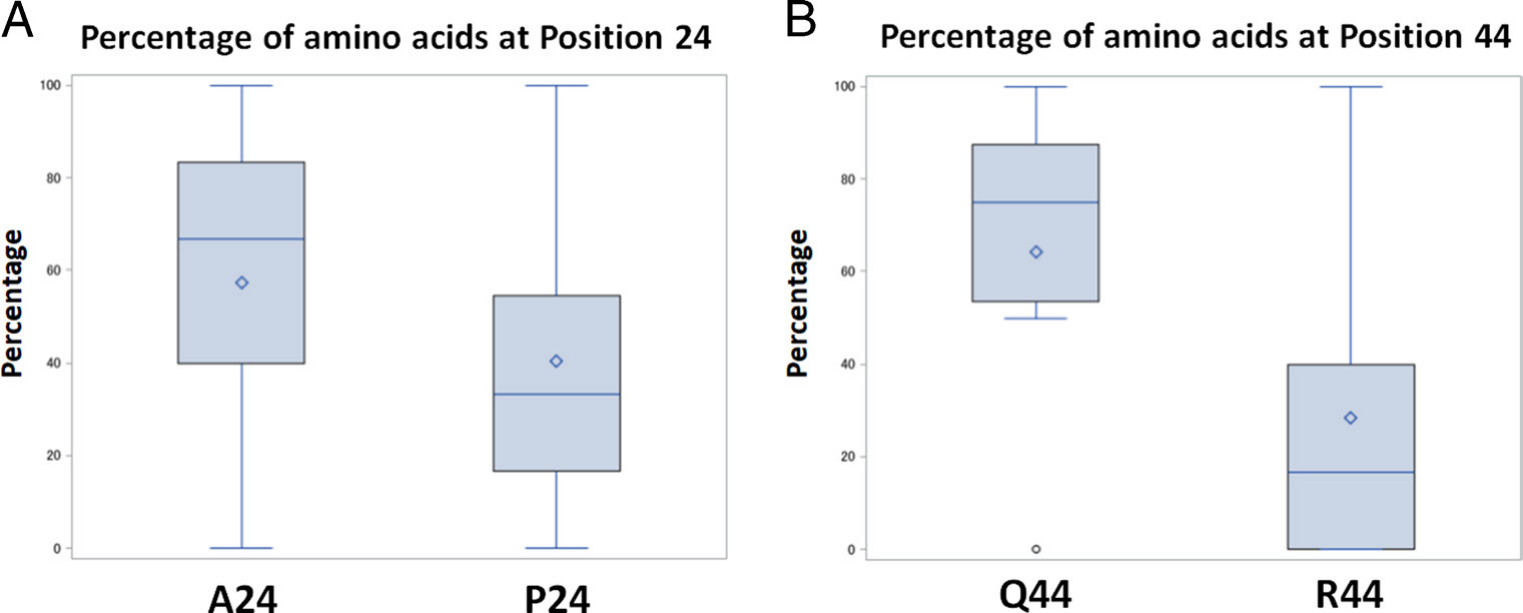
Box plots of *HgBioB* amino acid polymorphism trends among SCN field populations, showing the percentage of amino acid differences for A24P (A) and Q44R (B).

Since our previous studies were based on just two inbred SCN strains, we were interested to learn if sequence variation in the *HgBioB* gene was common in different field populations of SCN. To assess how common *HgBioB*-vir alleles are in different populations of SCN, the *HgBioB* gene was PCR-amplified and sequenced from a series of SCN populations. Sequencing analysis of four inbred SCN strains (OP25, OP20, OP50, and TN20) showed that these strains were more likely to be uniform (i.e., have 100% allelic frequencies) carrying single homozygous forms (Table 2). In contrast, sequencing results from 30 SCN field populations showed that in most SCN populations, the *HgBioB* gene was not present in a single homozygous form, but rather in a diverse mixture consisting of both SNPs resulting in both active and inactive forms of *HgBioB* (Table 2). Statistical analysis of the field sequencing data was conducted by generating a box plot and comparing the median values. This analysis showed that the *HgBioB*-vir alleles were present in a higher percentage than the *HgBioB*-avr alleles at both polymorphic regions (i.e., more A24 than P24; more Q44 than R44) (Fig. 2A,B, Table 3). This trend was also supported by conducting a Wilcoxon signed rank test, the nonparametric alternative to the paired *t*-test, which was chosen because the polymorphic nucleotide percentage data for avirulent and virulent alleles were not normally distributed. The data were considered paired since we compared the two amino acid forms at each polymorphic region (i.e., A24 vs P24 and Q44 vs R44). The Wilcoxon signed rank test results showed that *HgBioB*-vir allele ranks were significantly higher than *HgBioB*-avr allele ranks for both amino acid positions 24 (*Z*=105, *p*<0.05) and 44 (*Z*=121.5, *p*<0.01). Therefore, from both descriptive and inferential statistical results, we concluded that the virulent alleles are more prevalent than the avirulent alleles in the field sites tested.

**Table 2:** Sequencing analysis of *HgBioB* nematode populations showing the percentage of amino acids at each polymorphic region.

	24th Amino acid			44th Amino acid		
SCN populations	Threonine (**A**CT) %A	Alanine (**G**CT) %G	Proline (**C**CT) %C	Glutamine (C**A**A) %A	Arginine (C**G**A) %G	Proline (C**C**A) %C
Hg257		**100.00**		**100.00**		
Hg0-A		60.00	40.00	53.33	40.00	
Hg0-B	33.33	66.67		**100.00**		
OP 25^a^			**100.00**		**100.00**	
OP 20^a^			**100.00**			**100.00**
OP 50^a^	33.33		66.67			**100.00**
TN 20^a^		**100.00**		64.29	21.42	
Carbondale 6^b^		75.00	25.00	75.00	25.00	
Carbondale 30^b^			**100.00**		**100.00**	
C 104^c^	10.00	40.00	50.00	87.50	12.50	
C 432^c^		83.33	16.67	**100.00**		
EC 138^c^		66.67	33.33	83.33	16.67	
EC 146^c^		66.67	33.33	75.00	25.00	
EC 406^c^		60.00	40.00	75.00	25.00	
NC 228^c^		75.00	25.00	87.50		12.50
NC 238^c^		66.67	33.33	60.00	20.00	20.00
NC 305^c^		**100.00**		**100.00**		
NC 408^c^		50.00	50.00	60.00	40.00	
NE 336^c^		83.33	16.67	87.50	12.50	
NE 433^c^		**100.00**		**100.00**		
NW 138^c^		75.00	25.00	85.71	14.28	
NW 209^c^		57.14	42.85	66.67	33.33	
NW 308^c^		75.00	25.00	83.33	16.67	
NW 421^c^		66.67	33.33	83.33	16.67	
SC 349^c^		75.00	25.00	83.33	16.67	
SE 132^c^		60.00	40.00	66.67	33.33	
SE 148^c^			**100.00**		**100.00**	
SE 217^c^			**100.00**		**100.00**	
SW 207^c^		40.00	60.00	50.00	50.00	
SW 242^c^		45.45	54.54	60.00	40.00	
SW 251^c^		85.71	14.28	**100.00**		
WC 216^c^			**100.00**		**100.00**	
WC 229^c^		83.33	16.67	90.91	9.09	
WC 350^c^		90.90	9.09	**100.00**		

^a^Inbred SCN strains provided by Charlie Opperman (OP) and Terry Niblack (TN); ^b^SCN field populations from Carbondale, Illinois; ^c^SCN field populations provided by Gregory Tylka. The sampling of these populations was conducted in 2015 from different regions of Iowa. The geographical origins are indicated as follows: C = central; EC = east central; NC = north central; NE = northeast; NW = northwest; SC = south central; SE = southeast; SW = southwest; WC = west central; * Amino acids with 100 percent allelic frequencies are shown in boldface.

**Table 3:** Statistical analysis of amino acid percentages from nematode field populations.

	Amino acid position 24		Amino acid position 44	
	Alanine (A24)	Proline (P24)	Glutamine (Q44)	Arginine (R44)
Mean	57.28	40.46	64.07	28.47
SD	33.51	32.88	36.02	33.21
Median	66.67	33.33	75.00	16.67
Min	40.00	9.09	50.00	9.09
Max	100.00	100.00	100.00	100.00

**Figure 3:**
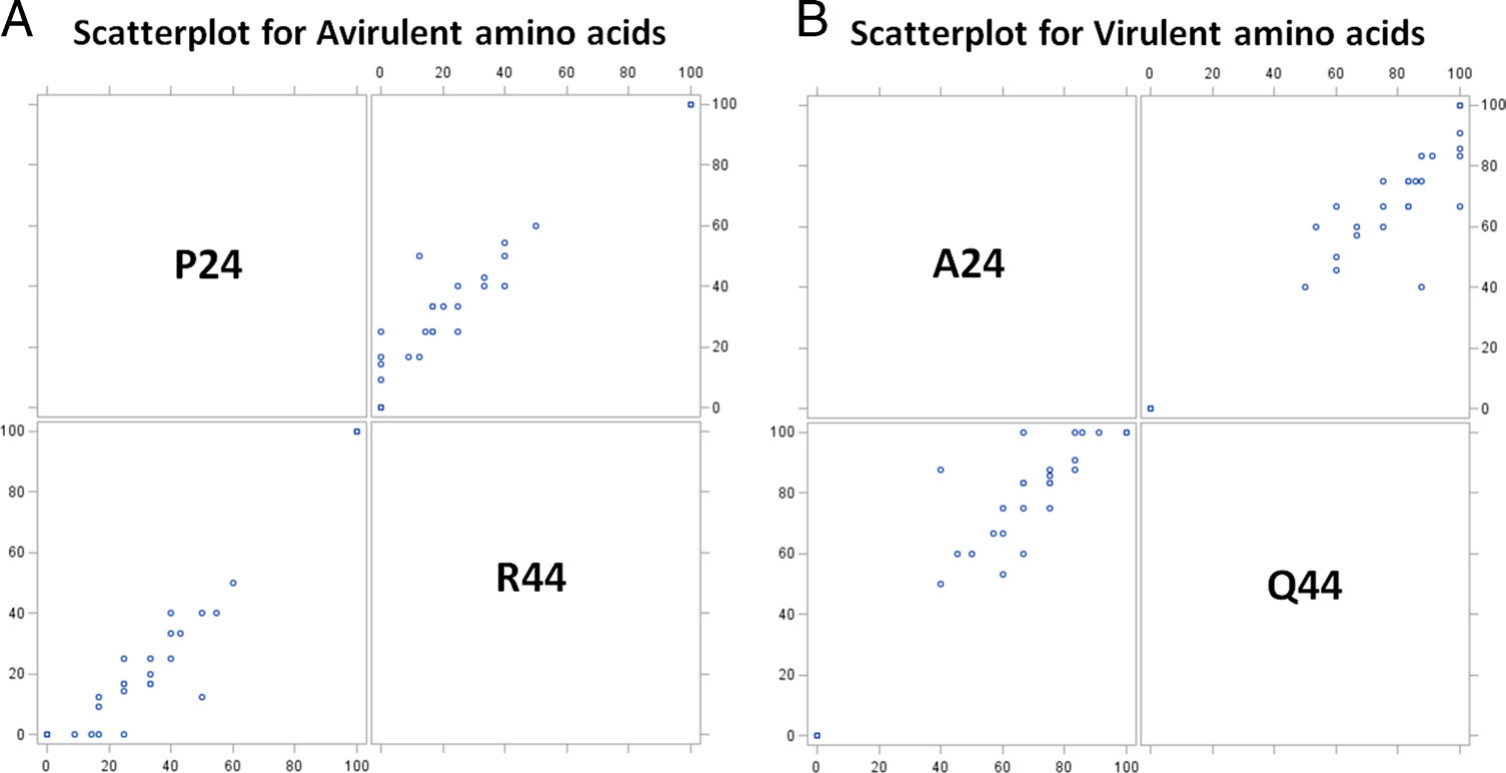
Scatterplots showing the *HgBioB* avirulent and virulent polymorphic amino acid pairs. (A) The avirulent pair shows a positive linear relationship between amino acid positions P24 and R44. (B) Similarly, the virulent pair also shows a positive linear relationship between amino acid positions A24 and Q44.

In addition, it is interesting to note that these SNP changes occur simultaneously in pairs, at about the same frequency, between amino acid positions 24 and 44. For example, if a SNP in *HgBioB* results in a P24 then it would be paired with an R44; if it were an A24 then it would be paired with a Q44. This linkage of SNP frequency was supported by testing the correlations between the virulent allele pair (A24 and Q44) as well as the avirulent allele pair (P24 and R44) using a Spearman rank-order correlation, the nonparametric alternative to the Pearson product-moment correlation, chosen because the assumptions of normality could not be met. The Spearman correlation results showed that there was a positive correlation for both virulent (*r*=0.8683, *n*=30, *p*<0.001) and avirulent (*r*=0.9184, *n*=30, *p*<0.001) pairs. Scatterplots for both avirulent and virulent pairs also summarize that there is a positive linear relationship between amino acid positions 24 and 44 (Fig. 3A,B). The strong, positive correlation between these positions suggests that these amino acid changes must both occur in the same protein to exert their desired, but unknown function. Although a rarity, alternative SNPs that result in a threonine at position 24 (T24) and a proline at position 44 (P44) were detected as well, suggesting an important but unknown function of these two amino acid positions in the *HgBioB* enzyme.

## Discussion

In this study, we compared the enzymatic activities of biotin synthase genes from virulent and avirulent inbred SCN strains and showed that the *HgBioB*-vir from virulent nematodes lacked activity while the *HgBioB*-avr from avirulent nematodes was fully active, suggesting *HgBioB* is important in SCN virulence. Biotin (vitamin B_7_ or vitamin H) is a member of the water-soluble vitamin B family and an essential enzyme cofactor, necessary for all three domains of life (Depeint *et al.*, 2006; Zempleni *et al.*, 2008; Waldrop *et al.*, 2012). This vitamin acts as a carboxyl carrier for biotin-dependent carboxylases, decarboxylases, and transcarboxylases, which are critical for fatty acid, amino acid, and carbohydrate metabolic processes (Knowles, 1989; Alban *et al.*, 2000; Attwood and Wallace, 2002; Cronan, 2014). Biotin is also involved in cell signaling, chromatin structure, and epigenetic gene regulation (Zempleni, 2005). More recent discovery shows that biotin plays a role in bacterial virulence (Feng *et al.*, 2014), supporting its potential importance in nematode virulence and parasitism.

Despite its crucial functions, the ability to synthesize biotin *de novo*, either completely or partially, is only found in bacteria, plants, and some fungi (Hall and Dietrich, 2007; Alban, 2011). Most of these organisms synthesize biotin from pimeloyl-CoA (ACP) in a highly conserved four-step pathway that consists of 7-keto-8-aminopelargonic acid, 7,8-diaminopelargnoic acid, DTB, and biotin (Streit and Entcheva, 2003; Alban, 2011; Lin and Cronan, 2011). However, animals do not have the ability to synthesize biotin (Hall and Dietrich, 2007) and must acquire vitamins from their diet (Craig *et al.*, 2008; Craig *et al.*, 2009). Nevertheless, it is strange that SCN, an animal and a parasite, lost the ability to synthesize biotin and then re-acquired the biotin synthase gene via HGT, because the nematode should be able to acquire biotin from its host. It should be noted that SCN does not have the complete biotin biosynthesis pathway, but a partial pathway that encodes the last key enzyme, biotin synthase (Craig *et al.*, 2009), which converts DTB to biotin (Cleary and Campbell, 1972). The presence of this incomplete *de novo* biotin biosynthesis pathway implies that biotin has an important function in SCN and suggests the nematode is scavenging DTB to make biotin from its host (Craig *et al*., 2009). Although free biotin can be present at high levels in plant cells, unlike animal cells which have more protein-bound biotin (Alban, 2011), it remains unknown if sufficient free biotin is available to SCN as they feed from syncytial cells of high metabolic activity. It is possible that SCN, a filter feeder that does not consume plants via chewing, cannot obtain enough biotin if it is bound to proteins. That is, their presumed prevalence for withdrawing nutrients via feeding tubes, which act as a molecular sieve (Böckenhoff and Grundler, 1994), may limit their vitamin ingestion; thus, having the biotin synthase gene, or other vitamin B biosynthetic genes, could be a selective advantage.

We previously predicted that virulent nematodes might gain a competitive advantage by producing a more enzymatically active biotin synthase (Bekal *et al.*, 2015) and utilizing the precursors to expand its salvage pathways (Craig *et al.*, 2008, 2009). In our complementation study, however, only the avirulent allele of *HgBioB* (*HgBioB*-avr) successfully complemented the growth of an *E. coli* strain containing a deletion of bioB. This showed that avirulent inbred SCN produces an active biotin synthase while virulent inbred nematodes contain an inactive form of the enzyme, thereby refuting our hypothesis.

However, it was possible that the inbred nature of the nematodes selected for a rare *HgBioB* allele that was inactive. To assess how common *HgBioB*-vir alleles are in natural populations of SCN, the *HgBioB* gene was PCR-amplified and sequenced from a series of nematode populations. The sequencing data showed that both SNPs were present in the field SCN populations, resulting in a mixture of both active and inactive forms of *HgBioB*, which is expected due to the heterozygous nature of most nematode populations. In most SCN populations, the virulent forms were prevalent, mirroring the increased prevalence of virulent SCN described by many nematologists (Niblack and Riggs, 2004; Mitchum *et al.*, 2007; Niblack *et al.*, 2008; Tylka and Mullaney, 2015). This implies that an opposing selection for *HgBioB* activity is occurring in the wild. It would be interesting to find a correlation, if any, between the prevalence of active/inactive forms of *HgBioB* with certain Hg-types in the field populations; however, we were not able to harvest enough cysts to carry out Hg-type bioassays and, therefore, all cysts that were harvested were used for the sequencing analysis. There also seems to be an important, but unknown reason why the SNP changes occur concurrently in pairs with similar frequencies between amino acid positions 24 and 44. Since the avirulent and virulent forms of the *HgBioB* alleles only differ at these two positions, we initially speculated that these amino acid sequence polymorphisms might affect the overall biotin synthase activity, especially if these SNPs were located close to the conserved regions responsible for substrate-binding or catalytic activity of the enzyme. However, these SNPs were not in the conserved C^53^xxxC^57^xxC^60^ motif (C, cysteine; x, any amino acid), a key characteristic of biotin synthase and other enzymes in the radical S-adenosyl-methionine superfamily (Frey and Booker, 2001; Jarrett, 2003; Berkovitch *et al.*, 2004). Both the presence of sequence polymorphisms at amino acid positions 24 and 44, and the evidence from the site-directed mutagenesis indicating the absolute requirement for both proline and arginine at those specific locations (P24 and R44) further stress the importance of these amino acids in spite of their unknown functional role in the *HgBioB* enzyme. Since our sequencing data showed that these SNPs did not occur randomly but changed in a coordinated fashion, these SNPs might lead to a complete change in protein function other than biotin synthase. Because the nematode encodes the last key enzyme involved in the biotin biosynthesis pathway, the nematode could have repurposed the biotin synthase enzyme for a completely different task. It has been reported that the housekeeping glutathione synthetase (GS) gene from *G. pallida* was repurposed to diversify its biochemical function, forming a novel class of GS-like enzymes (Lilley *et al.*, 2018). In a similar manner, it is possible that the biotin synthase gene may also modify its biochemical activity to produce a new family of biotin synthase-like enzymes for the evolution of its parasitic lifestyle.

It is also possible that these SNPs might convert the *HgBioB* enzyme into a biotin-related toxin-binding protein that may protect the virulent nematode from host defense mechanisms. Such biotin-related anti-metabolites do occur in bacteria (Hanka *et al.*, 1966, 1969, 1972), but it is unclear if such toxins play a role in SCN resistance. Regardless of the role the inactive form of *HgBioB* plays in the SCN life cycle, it would have to provide a survival advantage to the nematode to counteract any negative effects such as slower growth or lower reproduction due to the absence of biotin scavenging ability of an active *HgBioB*. It has been shown that a reproductive fitness cost is associated with virulence in *M. incognita* (Castagnone-Sereno *et al.*, 2007; Djian-Caporalino *et al.*, 2011), and therefore this could also be applicable to virulent SCN where low biotin availability could cause biotin deficiency, which may result in low reproductive fitness.

In the future, it would be interesting to see if virulent SCN also have a reproductive fitness cost by correlating a nematode’s biotin synthase activity or its biotin content with its reproductive index and/or cyst size. Further study of the biotin synthase genes from other SCN strains and even from other plant-parasitic nematodes may help elucidate how the loss of *HgBioB* activity might benefit SCN parasitism and/or virulence, which may lead to the development of novel methods to minimize soybean yield losses affected by these devastating pathogens.
